# Complete Genome Sequence of Methylococcus capsulatus MIR, a Methanotroph Capable of Growth on Methanol

**DOI:** 10.1128/mra.00542-22

**Published:** 2022-08-17

**Authors:** Igor Y. Oshkin, Ruslan Z. Suleimanov, Valentina N. Khmelenina, Andrey V. Mardanov, Nikolai V. Pimenov, Svetlana N. Dedysh

**Affiliations:** a Winogradsky Institute of Microbiology, Research Center of Biotechnology of the Russian Academy of Sciences, Moscow, Russia; b G. K. Skryabin Institute of Biochemistry and Physiology of Microorganisms, Scientific Center for Biological Research of the Russian Academy of Sciences, Pushchino, Moscow Oblast, Russia; c Institute of Bioengineering, Research Center of Biotechnology of the Russian Academy of Sciences, Moscow, Russia; University of Southern California

## Abstract

Methylococcus capsulatus MIR is an aerobic methanotroph that was isolated from an activated sludge sample and is capable of growth on methanol. The finished genome of strain MIR is 3.2 Mb in size. It encodes both MxaFI and XoxF methanol dehydrogenases, as well as three different isozymes of formate dehydrogenase.

## ANNOUNCEMENT

**FIG 1:**
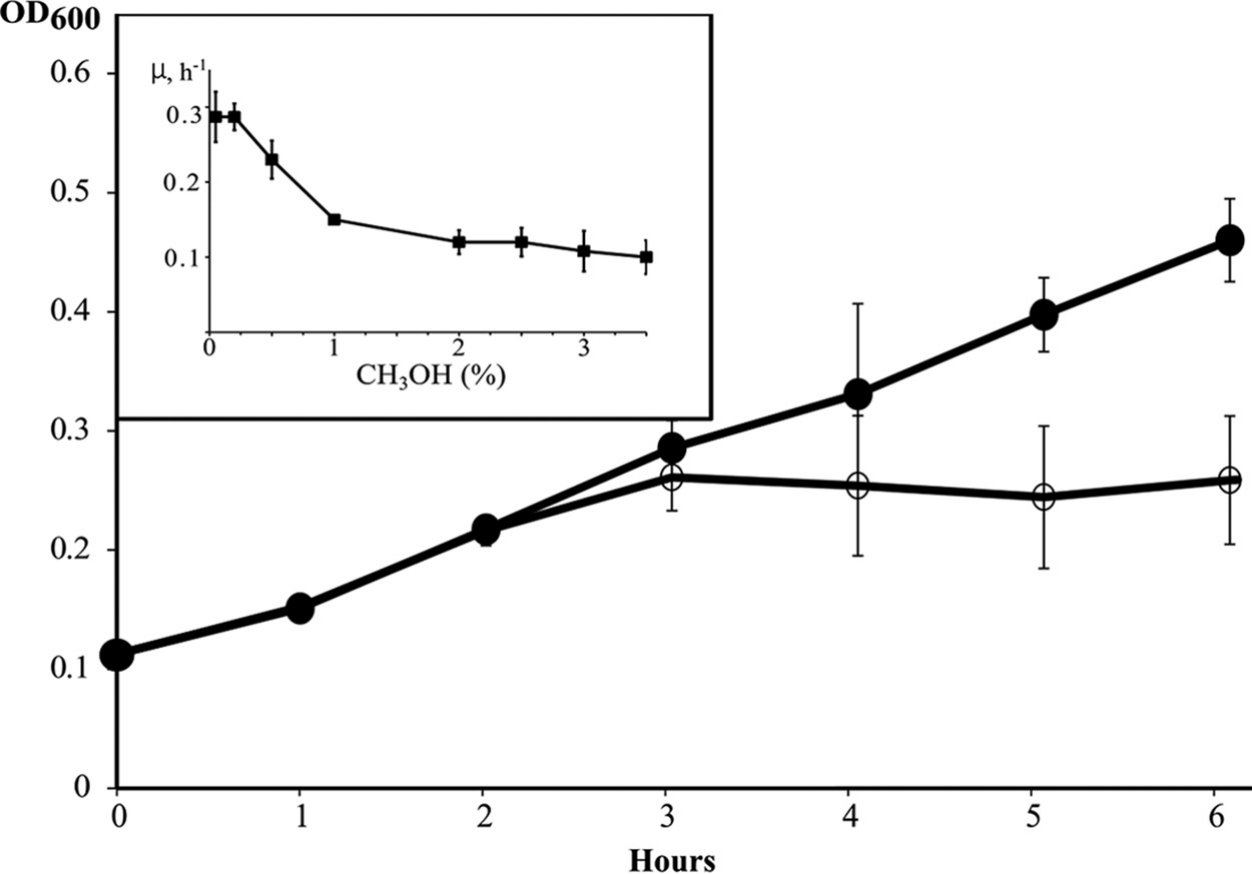
Growth of strain MIR on 20% (vol/vol) methane (filled circles) or 0.05% (vol/vol) methanol (empty circles). The inset shows specific growth rates being dependent on the methanol concentration (values calculated for a 3-h incubation period). All data are means of triplicates ± 1 standard error of the mean (shown by bars). Where error bars are not seen, they are contained within the symbol.

The species Methylococcus capsulatus represents aerobic thermotolerant methanotrophic bacteria that are widely distributed in various habitats (1, 2) and possess high biotechnological potential. With very few exceptions, characterized strains of this species display only trace growth on methanol (3). Here, we report the complete genome sequence of a new M. capsulatus isolate, strain MIR, which is capable of growth on methanol in the range of concentrations of 0.05 to 3.5% (vol/vol) in a mineral medium (3) during incubation at 42°C (Fig. 1). Strain MIR was isolated from the upper oxic layer of activated sludge from the Irkutsk municipal wastewater treatment plant using a mineral medium with 20% (vol/vol) methane and the previously described isolation procedure (3). Genomic DNA was extracted from a liquid culture of strain MIR grown with methane (3) using the standard cetyltrimethyl ammonium bromide (CTAB) and phenol-chloroform protocol (4). The 16S rRNA gene of strain MIR was PCR amplified using the primers 9f and 1492r (5) and displayed greatest similarity (99.93%) to that of M. capsulatus Bath (GenBank accession number AE017282.2).

The same DNA extract was used for genome sequencing by means of Illumina and Oxford Nanopore Technologies platforms. The shotgun genome library was prepared using the NEBNext Ultra II DNA library preparation kit (New England BioLabs, USA). The sequencing of this library on a MiSeq instrument (Illumina, San Diego, CA) generated 1,871,556 read pairs (2 × 300-nucleotide mode). Adapter removal and trimming of low-quality sequences (Q scores of <30) were performed using Cutadapt v3.4 (6) and Sickle v1.33 (https://github.com/najoshi/sickle), respectively. For Nanopore sequencing, the library was prepared using the 1D ligation sequencing kit (SQK-LSK109; Oxford Nanopore Technologies, UK). Sequencing of this library in an R9.4 flow cell (FLO-MIN106) using a MinION system yielded 230,762 reads, with a total length of 1,756 Mb. The raw read *N*_50_ value was 11,777 bp, the average read length was 7,609 bp, and the maximum read length was 114,186 bp. The Nanopore reads were demultiplexed and base called using Guppy v1.1. Hybrid assembly of short and long reads was performed using Unicycler v0.4.8 (7). Assemblies were evaluated with QUAST v5.0 (8) and BUSCO v5.1.2 (9). The final assembly represented the complete circular 3,187,097-bp genome, with 905× coverage. The assembled chromosome was annotated using the NCBI Prokaryotic Genome Annotation Pipeline (PGAP) (10) and Prokka (11). The default settings were used for all software.

In total, 2,859 protein-coding genes were predicted in the MIR genome. The genome contains two copies of the gene cluster encoding particulate methane monooxygenase (MMO) and one copy of the soluble MMO gene cluster. Both MxaFI and XoxF methanol dehydrogenases (12), as well as three different isozymes of formate dehydrogenase, are also encoded. Since C_1_ assimilation pathways are common among all *Methylococcus* species (2, 3), strain MIR may serve as a model organism for studying the metabolic basis of methanol tolerance.

### Data availability.

The whole-genome assembly of strain MIR has been deposited in DDBJ/ENA/GenBank under BioProject accession number PRJNA835301, BioSample accession number SAMN28099229, and SRA accession numbers SRX15161674 and SRX15161675. The version described in this paper is the first version, CP097161.1.
